# The association of communal vs. solitary dining on nutritional intake and depression risk in older adults: a systematic review and meta-analysis

**DOI:** 10.3389/fnut.2026.1878848

**Published:** 2026-07-13

**Authors:** Yingying Zhang, Yaqun Zhang, Jianzheng Cai, Weixia Yu, Ping Feng, Haifang Wang

**Affiliations:** 1 Department of Radiotherapy, The First Affiliated Hospital of Soochow University, Suzhou, China; 2 Department of Nursing, The First Affiliated Hospital of Soochow University, Suzhou, China; 3 Department of Critical Care Medicine, The First Affiliated Hospital of Soochow University, Suzhou, China

**Keywords:** commensality, depression, nutritional risk, older adults, social facilitation, solitary eating, systematic review

## Abstract

**Background:**

Global population aging and the transition toward empty-nest households have sharply increased solitary dining. This change in meal pattern is a key concern in geriatric health.

**Objective:**

To quantify the differences in dietary intake and depression risk between communal and solitary dining among community-dwelling older adults.

**Methods:**

Following PRISMA guidelines, we synthesized evidence from PubMed, Web of Science, Embase, and Cochrane Library from database inception through December 2025. Effect sizes were pooled using random-effects models. The PROSPERO registration of this systematic review and meta-analysis is CRD420251177507.

**Results:**

Of 12,088 records screened, 21 studies were included (11 Japan, 4 South Korea, 3 USA, 1 UK, 1 China, 1 Brazil, 1 Sweden; 16 high-quality, 5 moderate-quality per AHRQ). Compared to solitary dining, commensality is significantly associated with higher total energy intake (MD = 109.51 kcal, 17.39–201.62; *P* = 0.047), dietary fat intake (MD = 4.07 g, 95% CI 0.14–7.99, *P* = 0.0489), and meat/seafood consumption (MD = 21.28 g, 95% CI 2.27–40.29, *P* = 0.011). Solitary dining is a potent risk factor for depression (OR = 1.58, 95% CI: 1.33–1.87, *P* < 0.001). Across mealtimes, the highest depression risk is associated with solitary dinner (OR = 2.13, 1.57–2.89, *P* < 0.001).

**Conclusions:**

Communal dining is associated with a reduced risk of nutritional inadequacy and psychological distress through integrated mechanisms of social engagement and dietary diversification. Our findings support the inclusion of commensality as a strategic, non-pharmacological component in global geriatric health policy.

**Systematic review registration:**

https://www.crd.york.ac.uk/PROSPERO/view/CRD420251177507, identifier: CRD420251177507.

## 1 Introduction

Population aging is accelerating globally. The World Health Organization (WHO) estimates that by 2050, the global cohort of individuals ≥60 years old will constitute 22% of the total population, which is a nearly twofold increase compared to 2015 (1). This demographic transition is occurring alongside fundamental shifts in modern family structures, where “empty-nest” households and solitary living have transitioned from marginal occurrences to common social phenomena (2). The physical and psychological health of the geriatric population affects not only individual quality of life but also the social medical burden.

Dietary behavior, as a cornerstone of daily existence, serves a dual function: while providing the essential caloric and nutrient density required for physiological homeostasis (3), dietary behavior also exerts a profound influence on psychological wellbeing through its inherent social attributes (4, 5). Consequently, as the evolution of living arrangements and social environments sharply increases the incidence of eating alone among older adults (6), this lack of mealtime social companionship has emerged as a critical risk factor for numerous adverse health outcomes (7). The adoption of solitary eating is driven by a complex interplay of family fragmentation, living arrangements, diminished social competence, and declining health status. Mechanistically, eating alone drastically reduces social stimulation during mealtimes, which may not only be associated with adverse psychological outcomes such as clinical depression but also relate to a physiological decline in eating motivation, a phenomenon characterized by reduced appetite and diminished dietary diversity that escalates nutritional risk (8–11). For retired or widowed seniors, the loss of communal dining leads to a sharp reduction in the communicative value and hedonic pleasure of meals, which often parallels a weakening link between dietary patterns and overall health resilience (12).

In contrast, eating together, also referred to as commensality, is characterized by social interaction, a shared dining atmosphere, and collective food choices (13, 14). The social facilitation inherent in shared meals effectively prolongs meal duration and enhances eating motivation; it counteracts the appetite decline often induced by loneliness and correlates with superior nutritional status (13, 15, 16). Beyond physiological benefits, commensality provides older adults expanded social opportunities, more robust social support networks, and a stronger sense of belonging. These communal dining patterns are consistently linked to heightened levels of dining pleasure and improved psychological resilience, which reflects the critical linkage between social dietary behaviors and mental health outcomes (17, 18).

Despite these theoretical links, current empirical evidence remains fragmented and exhibits significant heterogeneity. While some studies report no significant association between eating alone and malnutrition in older adults (19), some studies have found that eating alone significantly predicts malnutrition risk and/or depressive symptoms, having controlled for potential confounders such as household income and self-care limitations (20, 21). Furthermore, inconsistencies in the definition of dietary patterns and the use of disparate outcome indicators across studies prevent consensus. To date, the lack of comprehensive systematic reviews and meta-analyses has made it difficult to synthesize the available evidence, quantify the magnitude of associations, or systematically identify the drivers of observed heterogeneity.

To address this gap, the present work synthesizes evidence from four major global databases to provide a quantitative assessment of (1) differences in food group, macronutrient, and total energy intake and (2) the risk of depression associated with disparate dietary patterns. These findings are intended to establish a robust evidence base for targeted dietary and psychosocial interventions aimed at optimizing geriatric health.

## 2 Methods

### 2.1 Search strategy

This study was designed and conducted in strict adherence to the Preferred Reporting Items for Systematic Reviews and Meta-Analyses (PRISMA) Statement (22). The search strategy was constructed based on a combination of Medical Subject Headings (MeSH) terms and free-text keywords. The syntax was adjusted according to the specific rules of the four databases (PubMed, Web of Science, Embase, and the Cochrane Library). The time span was from database inception to December 2025. Supplementary Tables S1–S4 describes the search strategy in detail.

### 2.2 Inclusion and exclusion criteria

This meta-analysis of observational studies did not involve intervention or control group designs. Therefore, the PEOS framework was adopted. Studies meeting the following Population, Exposure, Outcome, and Study design criteria were included:

- Population (P): Older adults aged ≥60 years;
- Exposure (E): Eating alone / eating together (distinguished from social isolation, or other irrelevant indicators);
- Outcomes (O): Quantifiable dietary outcomes (total energy intake, macronutrient intake, or food group intake) or quantifiable psychological outcomes (depression);
- Study Design (S): Cross-sectional study.

Studies were excluded if study participants were < 60 years old or had severe dementia, advanced diseases, or acute hospitalization. Studies were also excluded if they

- did not separately report data on older adults;
- failed to distinguish between eating alone/eating together;
- did not clearly define exposure or outcome indicators;
- only reported indicators other than dietary or psychological outcomes;
- did not report sufficient raw data for meta-analysis;
- were not a cross-sectional study (reviews, meta-analyses, commentaries, animal experiments, conference abstracts; randomized controlled trials, case-control studies, case reports, etc.).

### 2.3 Data extraction

Data extraction was performed independently by two reviewers using a standardized form; discrepancies were resolved through consensus or arbitration by a third investigator. The following information was extracted from each included study: (1) basic characteristics, including first author, publication year, and country; (2) participant characteristics, including sample size, setting, and age; (3) exposure and outcomes, including the definitions and measurement methods of eating alone/together, the assessment tools, and the effect sizes (e.g., OR, with 95% CI). To minimize potential bias from confounding factors, we prioritized extracting fully adjusted effect sizes such as adjusted odds ratios, particularly those controlling cohabitation status or living arrangements, to better isolate the specific impact of eating alone. To maximize data inclusion, if a study did not report adjusted data, crude unadjusted estimates were utilized for pooling.

### 2.4 Quality assessment

The quality of the included studies was assessed using the Observational Study Quality Assessment Scale developed by the Agency for Healthcare Research and Quality (AHRQ) (23). This tool was selected because all 21 primary studies included in this study strictly utilized a cross-sectional design, making the 11-item AHRQ scale the most methodologically appropriate instrument validated for quantifying the risk of bias in cross-sectional data. This scale consists of 11 items covering core dimensions such as study population selection, exposure measurement, outcome measurement, confounding control, and statistical analysis. Each item was scored as 1 point for “yes,” 0 point for “no,” and 0 point for “unclear” (item 5 was reverse-scored). The total score ranged from 0 to 11. The grading criteria were: 0–3 points, low quality, 4–7 points, moderate quality, 8–11 points, high quality.

### 2.5 Statistical analysis

Statistical analysis was performed using the meta and metafor packages in R 4.5.0. The heterogeneity between studies was assessed via Cochran's Q test (24) and quantified using the I^2^ statistic as follows: < 25%, low heterogeneity; 25%−75%, moderate heterogeneity; >75%, high heterogeneity. Because of the anticipated methodological and clinical heterogeneity across studies, a random-effects model was employed for data pooling (25). For continuous outcome indicators (e.g., food intake, nutrient intake, energy intake), the pooled effect size was expressed as the mean difference (MD) with 95% confidence interval (CI); for dichotomous outcome indicators (e.g., depression incidence rate), the odds ratio (OR) with 95% CI was used. Additionally, sensitivity analysis was conducted by sequentially omitting one study at a time to evaluate the stability of the pooled results. In accordance with the Cochrane Handbook guidelines, outcomes involving ≥10 studies were set for publication bias assessment using visual inspection of funnel plot.

## 3 Results

### 3.1 Literature search and screening results

**Figure 1:**
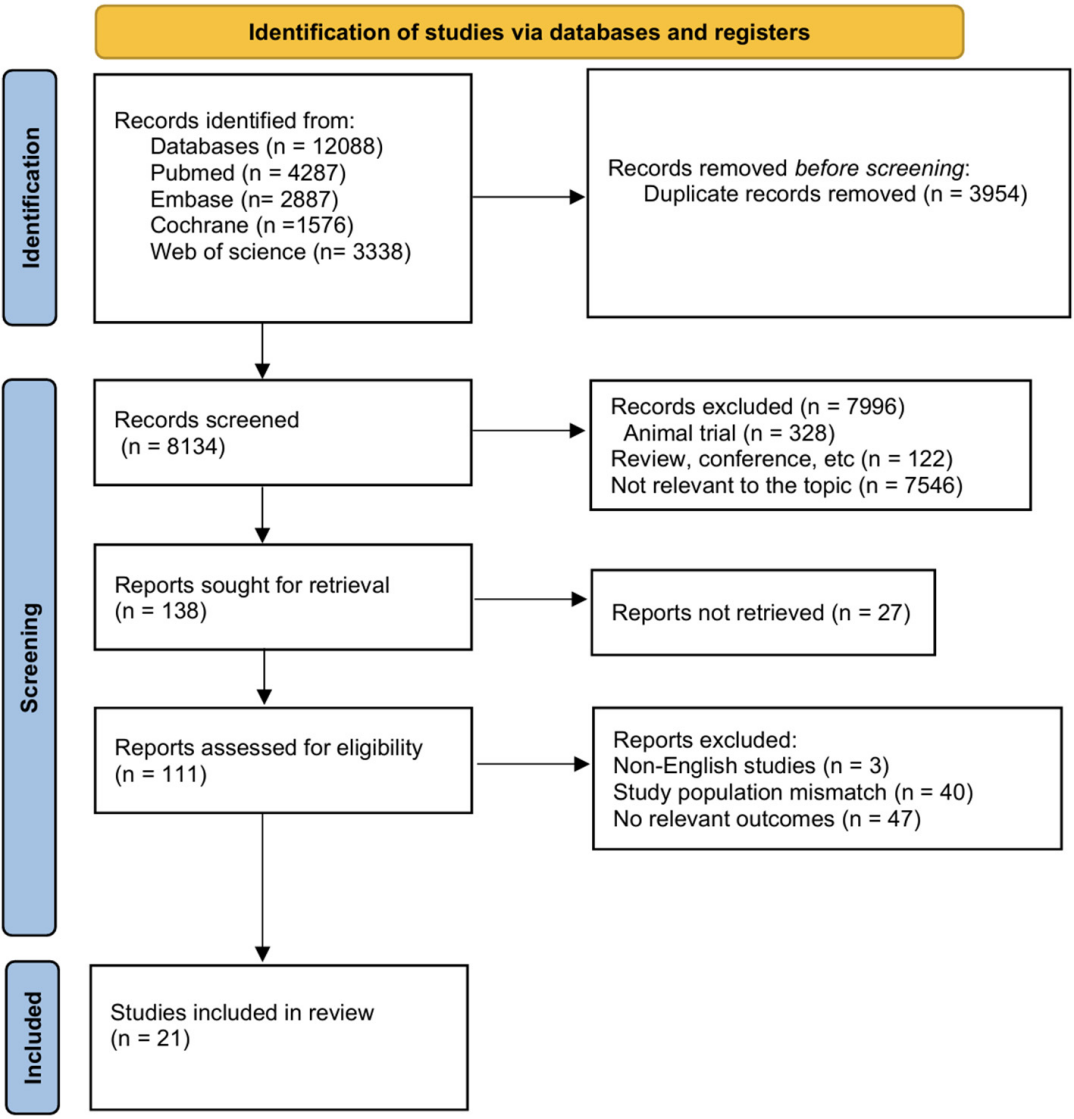
Retrieval, screening, and inclusion of studies.

A total of 12,088 articles were initially retrieved from four major databases (Figure 1), and 8,134 articles remained after algorithmic deduplication using EndNote. During the title and abstract screening (*n* = 8,134), initial disagreements between the two independent reviewers occurred for only 22 records (0.27%); among these, 18 records were resolved through consensus discussion, and the 4 remaining records were arbitrated by a third reviewer (Cohen's κ = 0.89). This primary screening eliminated 328 animal experiments, 122 review articles, and 7,546 studies irrelevant to the research topic, leaving 138 articles for full-text review. After excluding 27 reports with full texts unavailable, the remaining 111 articles underwent rigorous full-text review. At this assessment stage (*n* = 111), initial disagreements occurred for 5 reports (4.50%), of which 3 reports were resolved by re-evaluating the original full texts and consensus discussions, while the other 2 reports were arbitrated by a third reviewer (Cohen's κ = 0.84). In these articles, 3 were non-English, 27 did not have available full texts, 40 had the wrong population, and 47 did not have complete data. Finally, 21 studies were retained, all of which were cross-sectional studies. Table 1 summarizes the 21 studies that met the inclusion criteria (7, 9, 21, 26–43).

**Table 1:** Characteristics of included studies.

References	Participants^†^	Exposure^†^	Assessment^§^	Primary outcome^†‡^
Yamaguchi et al. (26), Japan	CDOA (*n* = 1,101) 71.6 ± 4.8 years	Eating A/T (breakfast/dinner)	FI: FFQ PS: K6 scale	• Alone × Low n-3 PUFA (vs. Social × High): 2.18 (1.05–4.55) • K6 score ≥5: 30.0% (303/1011)
Tani et al. (7), Japan	CDOA (*n* = 82,364) ≥65 years	Eating A/T	FI: SRQ	• A/T in males: Low vegetable/fruit. 1.59 (1.47–1.72), underweight 1.22 (1.02–1.45)
Pinheiro et al. (27), Brazil	CDOA (*n* = 8,336) ≥60 years	Living A/T	FI: R24h	• Living A/T: Low solid fats 0.76 (0.66–0.88); Low leafy veg 0.72 (0.62–0.83)
Kimura et al. (28), Japan	CDOA (*n* = 856) 76.6 ± 7.8 years	Eating A/T	FI: FDSK-11 PS: GDS-15	A/T: • FDSK-11: 9.9 ± 1.3 vs. 10.2 ± 1.3 (*P* = 0.002) • GDS-15: 5.7 ± 4.3 vs. 4.4 ± 3.8 (*P* < 0.001) • BMI (men): Alone < Together (*P* < 0.05)
Björnwall et al. (29), Sweden	CDOA (*n* = 695) 70–75 years	Eating A/T	FI: food index; R24h	• A/T: Low vegetable intake 0.68 (0.48–0.95); No difference in food index 1.03 (0.72–1.47)
Kwon et al. (30), South Korea	CDOA (*n* = 4,069) ≥65 years	Eating alone (vs. together 3/d)	FI: R24h; FFQ PS: PHQ-9; EQ-5	• A/T: PHQ-9 ≥10, 2.464 (1.527–3.975); EQ-5, 0.773 vs. 0.820
Minagawa-Watanabe et al. (31), Japan	CDOA (*n* = 993) 64–65 years	Dining with ≥2 companions (vs. alone)	FI: FFQ PS: GDS-15	• Alone (Ref) vs. ≥2 companions: β for Energy 143.85 (30.05–257.65), Protein 6.32 (1.05–11.59), Fat 6.78 (2.44–11.12), Carb 17.43 (1.48–33.37); GDS 28.4% vs. 24.3% (*P* = 0.01)
Ishikawa et al. (32), Japan	CDOA (*n* = 2,196) ≥65 years	Eating together (vs. alone < 1/month	FI: FDI PS: KYC	• A/T: Lower food diversity 46.7% vs. 37.1%(*P* < 0.001); Lower subjective health 33.3% vs. 13.5% *(P* < 0.001); Non-frail (Men): 58.2% vs. 85.0%; OR 0.48 (0.35–0.68)
Yokoro et al. (33), Japan	CDOA (*n* = 1,780) 73.9 ± 6.0 years	Living A/T	FI: DVS PS: FCI-5	• A/T in men: Frailty 1.8 ± 1.2 vs. 1.3 ± 1.1 (*P* < 0.001); Dietary variety 2.8 ± 2.0 vs. 3.8 ± 2.1 (*P* < 0.001)
Ishida and Ishida (34), Japan	CDOA (*n* = 1,336) ≥65 years	Eating A/T	FI: a 5-item questionnaire	• Single men (vs. women): Y1, −0.213; Y2, −0.352; Y3, −0.187; Y4, −0.189; Y5, −0.160
Locher et al. (35), USA	CDOA (*n* = 50) ≥60 years	Eating A/T	FI: R24h	• A/T: Caloric intake/meal −114.0 kcal (*P* = 0.009)
Takiguchi et al. (36), Japan	CDOA (*n* = 51) ≥65 years	Eating A/T, women only	FI: DRs2d	• A/T in Women: Fresh fruit 151.2 ± 81.6 vs. 97.7 ± 62.9 g (*P* < 0.05)
Prothro et al. (37), USA	CDOA (*n* = 95) ≥65 years	Living A/T	FI: DRs2d; R24h	• A/T: PTER 75.0 ± 27.8 vs. 74.5 ± 22.8 (NS); Carb (% kcal) *t* = 2.72 (*P* < 0.01); Fat (% kcal) *t* = −2.65 (*P* < 0.01)
Son et al. (38), South Korea	CDOA (*n* = 3,729) ≥65 years subgroup	Eating alone (vs. together 3/d)	PS: MHS	• Alone × High depression. (vs. Social × Low depression.): Men 1.72 (1.27–2.34); Women 1.58 (1.28–1.95)
Kang et al. (39), South Korea	CDOA (*n* = 4,959) 72.9 ± 0.1 years	Eating alone (vs. together 3/d)	PS: SRQ	• Alone × High depression/anxiety (vs. Family 3/d × Low depression/anxiety): aOR 0.72 (0.58–0.89)
Kuroda et al. (40), Japan	CDOA (*n* = 1,856) ≥65 years	Eating A/T	PS: GDS-15	• Alone × High depressive symptoms (vs. Together × Low): 2.96 (1.8–5.0)
Kantilafti et al. (41), Cyprus	CDOA (*n* = 248) median 72 years	Eating A/T	FI: MNA PS: GDS-15	• Alone × High depression (vs. Together × Low): aOR 2.00 (1.00–3.99)
Wang et al. (42), China	CDOA (*n* = 7,968) median 67 years	Eating A/T	PS: PHQ-9	• A/T: Depressive symptoms 12.9% vs. 4.5%−9.4% (*P* < 0.001)
Kushida et al. (43), Japan	CDOA (*n* = 2,764) ≥65 years	Eating alone at all meals (vs. Never)	PS: GDS-5	• Alone × High depression (vs. Together × Low): Breakfast 1.71 (1.40–2.09); Lunch 1.43 (1.17–1.76); Dinner 2.13 (1.57–2.90)
Sakurai et al. (9), Japan	CDOA (*n* = 710) 73.8 ± 5.5 years	Eating alone (vs. Together ≥1/d)	PS: SDS	• A/T: Higher SDS score, main effect significant (*P* < 0.001)
Moon et al. (21), South Korea	CDOA (*n* = 2,702) ≥60 years	Eating A/T (≤ 2/d vs. >2/d)	PS: SGDS-K	• Alone × High depression (vs. Together × Low) in Robust: Living together 1.60 (1.21–2.00); Living alone 1.94 (1.46–2.56)

† CDOA, community-dwelling older adults. The age of the study participants is described using mean ± standard deviation, median, or range as appropriate. A/T, alone vs. together.

§ FI, food intake; PS, psychological status. DRs2d, 2-day dietary records; DVS, dietary variety score; EQ-5D, Euro Quality of Life five-dimension; FCI-5, 5-item frailty screening index; FDI, food diversity index; FDSK-11, 11-item food diversity score Kyoto; FFQ, food frequency questionnaire; GDS-15, 15-item geriatric depression scale; GDS-5, Japanese version 5-item geriatric depression scale; K6 scale, Kessler 6 scale; KYC, Kaigo-Yobo checklist; MHS, mental health survey; MNA, mini nutritional assessment; PHQ-9, patient health questionnaire-9; R24h, 24-h food recall; SDS, Zung self-rating depression scale; SGDS-K, Korean version of the 15-item geriatric depression scale; SRQ, self-reported questionnaires.

‡ Reported as odds ratio or mean difference with 95%CI as appropriate.

### 3.2 Basic characteristics of included studies

All included studies were published before December 2025 from 7 countries: Japan, 11; South Korea, 4; USA, 3; UK, 1; China, 1; Brazil, 1; Sweden 1. Various assessment tools were used, and the most frequently used too for assessing dietary intake and depression were 24-h dietary recall (5 studies) and GDS (multiple versions, 5 studies), respectively. Among them, 13 studies provided sufficient and comparable quantitative data suitable for the meta-analysis, while the remaining 8 studies were only synthesized qualitatively due to the lack of extractable mean values and standard deviations or standard effect sizes.

Regarding the 8 studies excluded from the quantitative synthesis, specific technical or data constraints precluded their inclusion in the meta-analysis. Specifically, Ishida et al. (34) reported only non-standard multiple regression coefficients (Y1–Y5) rather than raw dietary intake statistics. Yokoro et al. (33) and Ishikawa et al. (32) utilized specialized dietary diversity metrics, specifically the DVS and FDI, which could not be statistically pooled with the standardized food group weights (g/day) from other studies. The remaining reports excluded studies failed to report continuous consumption values (Mean ± SD) or standard errors required for random-effects model pooling, and were thus retained exclusively for qualitative systematic description.

### 3.3 Quality assessment

The AHRQ Observational Study Quality Assessment Scale was used to evaluate the quality of the included studies. Table 2 presents the scoring results.

**Table 2:** Quality assessment results.

References	1	2	3	4	5	6	7	8	9	10	11	Total score
Yamaguchi et al. (26)	1	1	1	1	1	1	1	1	0	0	0	8
Tani et al. (7)	1	1	1	1	1	0	1	1	0	1	0	8
Pinheiro et al. (27)	1	1	1	1	1	1	1	1	0	0	0	8
Kimura et al. (28)	1	1	1	1	1	0	1	1	0	1	0	8
Björnwall et al. (16)	1	1	1	1	1	1	1	1	0	1	0	9
Kwon et al. (30)	1	1	1	1	1	1	1	1	0	1	0	9
Minagawa-Watanabe et al. (31)	1	1	1	1	1	0	1	1	0	1	0	8
Ishikawa et al. (32)	1	1	1	1	1	0	1	1	0	1	0	8
Yokoro et al. (33)	1	1	1	1	1	0	1	1	0	1	0	8
Ishida and Ishida (34)	1	1	1	1	1	0	0	1	0	1	0	7
Locher et al. (35)	1	0	0	0	1	0	1	1	0	1	0	5
Prothro et al. (37)	1	1	0	0	1	1	1	1	0	1	0	7
Takiguchi et al. (36)	1	1	1	0	1	1	1	1	0	1	0	8
Son et al. (38)	1	1	1	1	1	1	1	1	0	1	0	9
Kuroda et al. (40)	1	1	1	1	1	0	1	1	0	1	0	8
Kang et al. (39)	1	1	1	1	1	0	1	1	0	0	0	7
Kantilafti et al. (41)	1	1	1	0	1	1	1	1	0	1	0	8
Wang et al. (42)	1	1	1	1	1	1	1	1	0	1	0	9
Kushida et al. (43)	1	1	1	0	1	1	1	1	0	1	0	8
Sakurai et al. (9)	1	1	1	0	1	1	1	1	0	1	0	8
Moon et al. (21)	1	1	1	1	1	1	1	1	0	1	0	9

### 3.4 Outcomes

#### 3.4.1 Food intake

**Figure 2:**
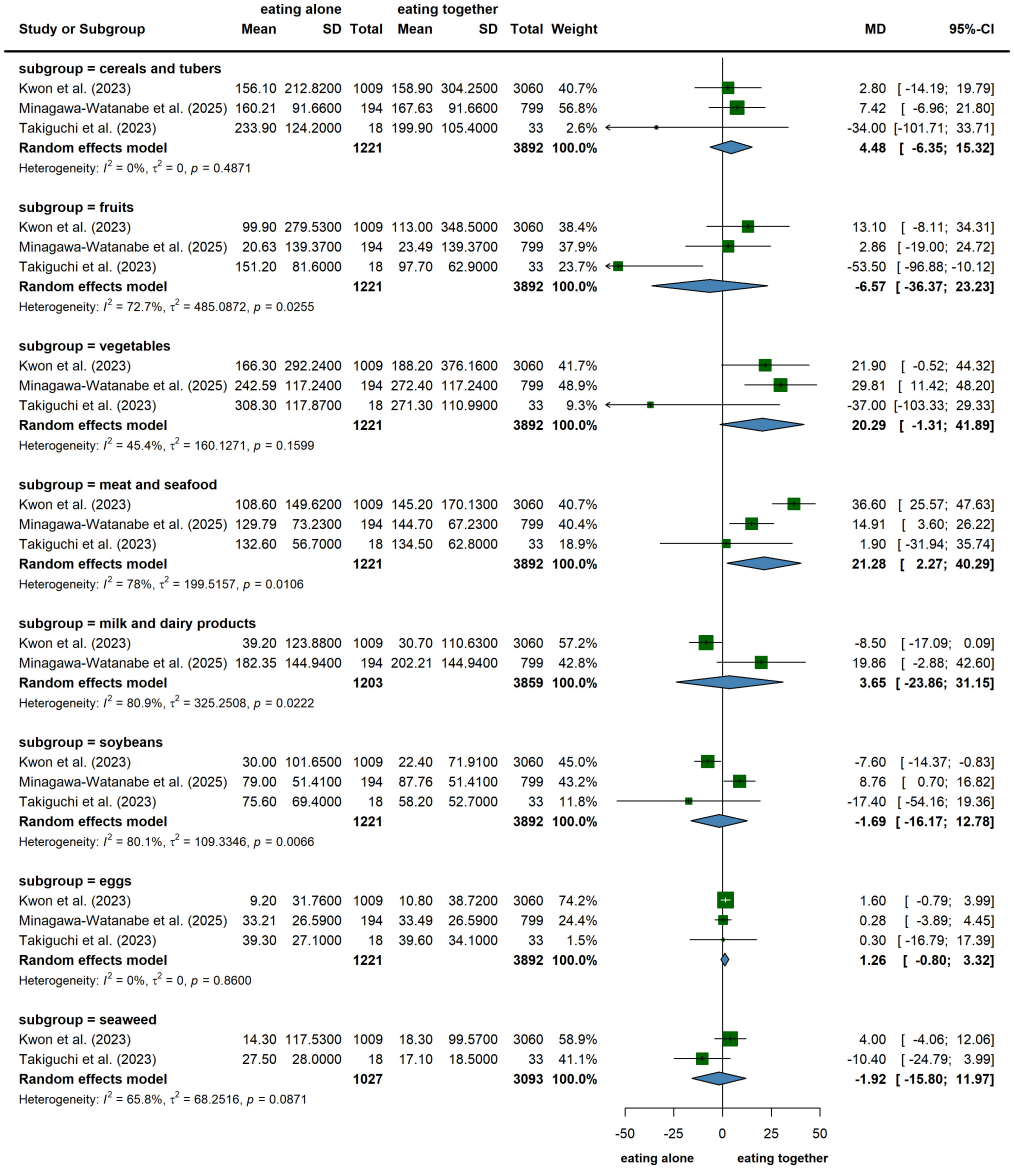
Differences in food intake between older adults eating alone and eating together.

Nine studies investigated the associations of eating alone/together on food intake (7, 27, 29–34, 36), among which three provided detailed data eligible for meta-analysis (30, 31, 36). A total of 8 food subgroups were included, and effect sizes were pooled using a random-effects model (Figure 2). The pooled MD in food intake between eating alone and eating together ranged from −6.57 to 21.28 g/day across subgroups. Only the meat and seafood subgroups showed a statistically significant higher intake in the eating together group (MD = 21.28 g/day, 95% CI: 2.27–40.29), and no significant differences were found in other subgroups.

#### 3.4.2 Macronutrient intake

**Figure 3:**
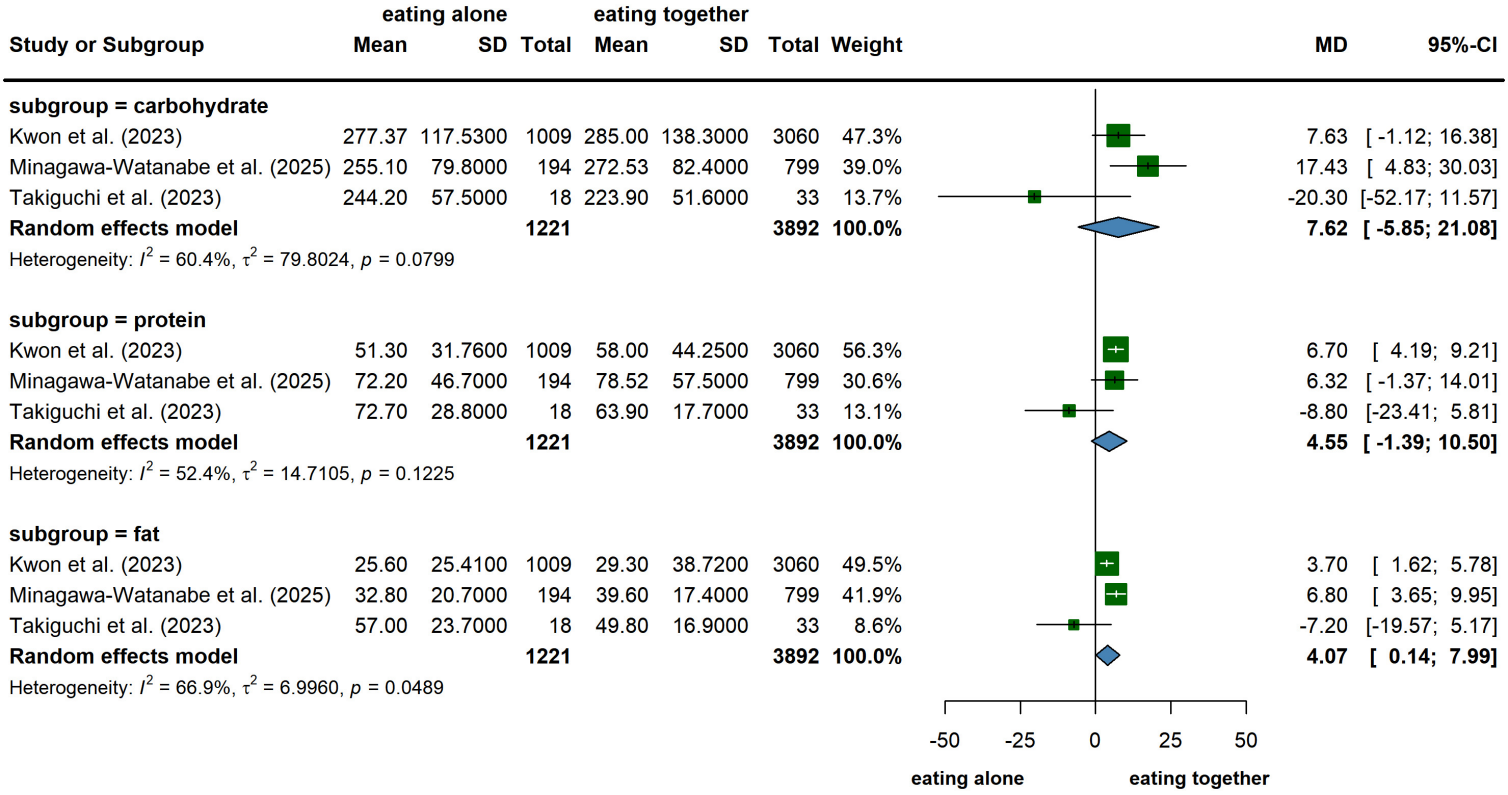
Differences in total macronutrient intake between older adults eating alone and together.

Four studies investigated the associations of eating alone or eating together on macronutrient intake (30, 31, 36, 37), among which three were included in a meta-analysis (30, 31, 36). The random-effects meta-analysis (Figure 3) showed that the pooled MD for fat intake was 4.07 (95% CI: 0.14–7.99, *I*^2^ = 66.9%, *P* = 0.0489), indicating significantly higher fat intake in older adults eating together. No significant differences were found for the intake of carbohydrate (MD = 7.62, 95% CI: −5.85 to 21.08, *I*^2^ = 60.4%, *P* = 0.0799) or protein (MD = 4.55, 95% CI: −1.39 to 10.50, *I*^2^= 52.4%, *P* = 0.1225) between eating alone and eating together.

#### 3.4.3 Total energy intake

**Figure 4:**
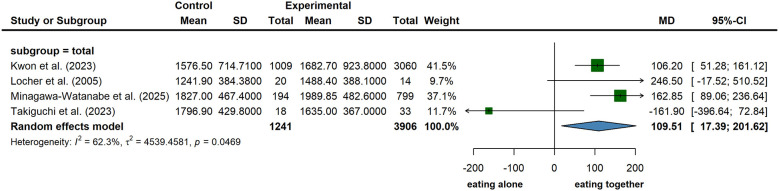
Differences in total energy intake between older adults eating alone and together.

Five studies reported energy intake (30, 31, 35–37), among which four were included in a meta-analysis (Figure 4). The pooled MD was 109.51 (95% CI: 17.39–201.62, *I*^2^ = 62.3%, *P* = 0.0469).

#### 3.4.4 Psychological status

**Figure 5:**
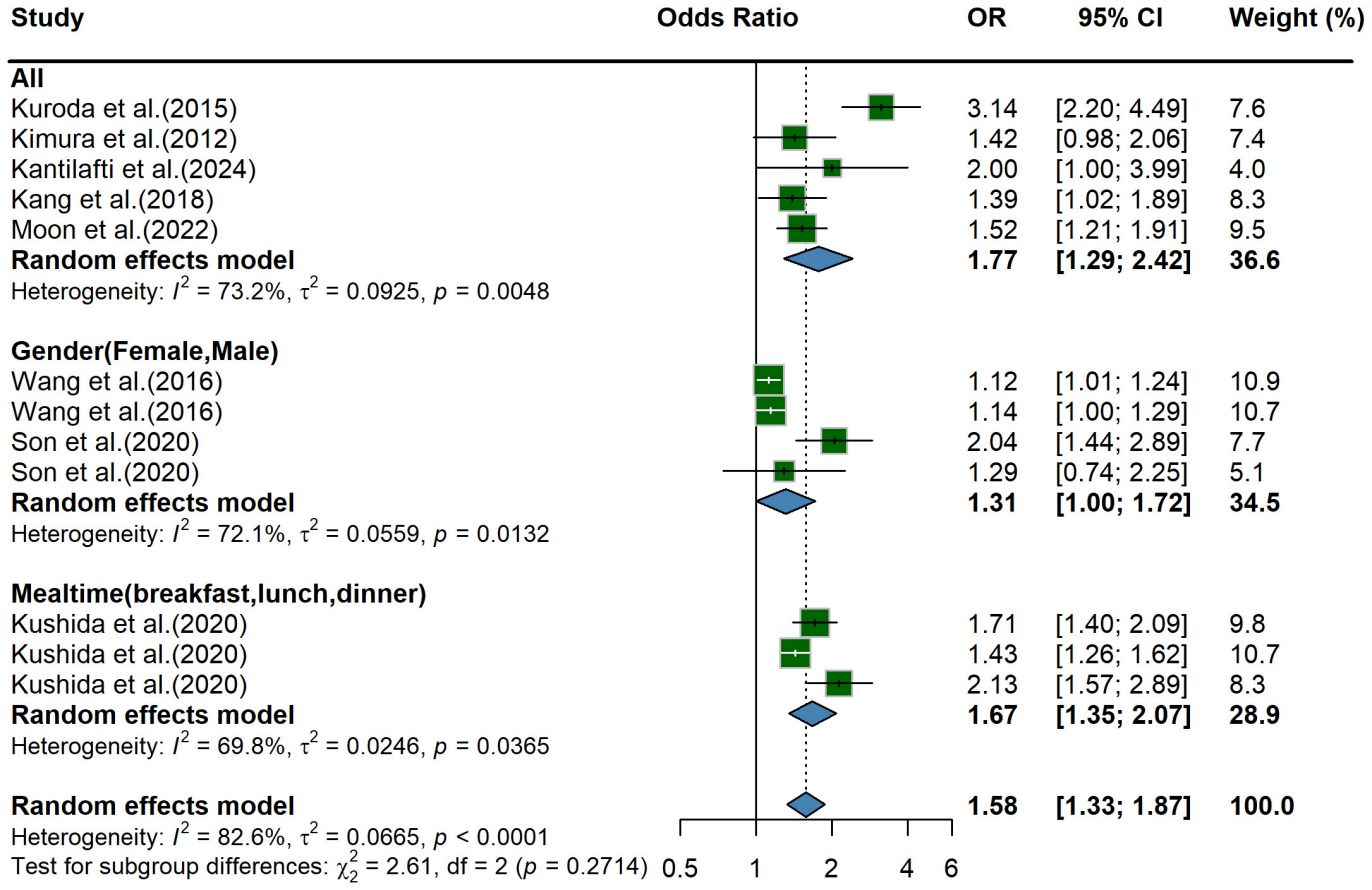
Differences in psychological status between older adults eating alone and together.

Nine studies reported adverse psychological outcomes (depression) (9, 21, 28, 38–43), and eight of them (21, 28, 38–43) were included in a meta-analysis (Figure 5). Eating alone was significantly associated with an elevated risk of adverse psychological events, with an overall pooled OR of 1.58 (95% CI: 1.33–1.87, *P* < 0.0001), but high heterogeneity was observed among the studies (*I*^2^ = 82.6%, *P* < 0.0001). Subgroup analyses indicate the association across genders was marginally significant (OR = 1.31, 95% CI: 1.00–1.72). This risk persisted across mealtimes (pooled OR = 1.67, 95% CI: 1.35–2.07), with eating dinner alone carrying the highest risk (OR = 2.13, 95% CI: 1.57–2.89).

Subgroup analyses, stratified by study quality, country, and psychological assessment scale, were performed to explore the sources of high heterogeneity for psychological outcomes (Supplementary Figures S1–S3). While subgrouping by study quality did not yield statistically significant differences in the pooled effect sizes across groups (*P* = 0.446), statistically significant between-subgroup differences were observed for country (*P* < 0.0001) and type of psychological assessment scale (*P* = 0.0404). A stronger association between solitary eating and the risk of adverse psychological outcomes was found in the Japanese population (OR = 1.84, 95% CI: 1.40–2.42, *I*^2^ = 80.7%). Notably, heterogeneity decreased substantially in the Korean (OR = 1.56, *I*^2^ = 9.5%) and Chinese (OR = 1.13, *I*^2^ = 0%) subgroups after stratification. However, the results for the Chinese demographic must be interpreted with caution, as they are drawn from a single large-scale cross-sectional study. Furthermore, studies utilizing the GDS scale reported higher risks (OR = 1.78, *I*^2^ = 72.0%) than those using other measurement tools (OR = 1.31, *I*^2^ = 66.5%). These findings indicate that regional and cultural discrepancies, as well as differences in the measurement tools for psychological outcomes, are the primary sources of heterogeneity in the meta-analyses.

### 3.5 Publication bias assessment

No quantitative assessment of publication bias (e.g., funnel plots or Egger's test) was performed for the pooled dietary or psychological outcomes. In strict accordance with the Cochrane Handbook guidelines, such statistical assessments are only methodologically reliable when a specific pooled category comprises a minimum of 10 primary studies (44). Because all our quantitative dietary and macronutrient subgroups contained fewer than 10 studies, funnel plot asymmetry testing was omitted to avoid underpowered and unreliable interpretations.

### 3.6 Sensitivity analysis

To verify the robustness of the pooled effect sizes for each outcome, leave-one-out meta-analysis was performed for the six food category subgroups, three macronutrients, the total energy intake, and the risk of adverse psychological status. The analyses involved sequentially deleting one study, refitting the random-effects model, and evaluating the changes in the direction, range, and statistical significance of effect sizes (Supplementary Figures S4–S14). For the risk of adverse psychological events remained highly robust, no single study exerted a disproportionate influence on the pooled estimates. For most food categories and the carbohydrate macronutrient subgroup, there was minimal interference from heterogeneity. In contrast, significant fluctuations were observed for total energy intake, protein, fat, and vegetable subgroups, indicating lower stability. In those instances, heterogeneity must be primarily driven by small-sample outliers and heavily weighted large-scale studies, and the corresponding results should be interpreted with caution. However, the core findings of this study were still corroborated by the sensitivity analyses.

## 4 Discussion

In this work, we synthesized 21 cross-sectional studies to provide a robust synthesis of the association of dining patterns, specifically solitary vs. communal eating, on the dietary habits and psychological health of older adults. The pooled estimates demonstrate that solitary eating is associated significantly with a reduction in total caloric intake, dietary fat, and the consumption of high-quality protein sources such as meat and seafood, as well as an elevation in the risk of adverse psychological outcomes. These findings verify the connection between dietary environment and geriatric health and offer key evidence for the development of targeted public health interventions.

Commensality appears to be strongly associated with nutritional balance in the geriatric population (13, 18, 29, 45), and the underlying mechanism is multifaceted. First, communal dining typically involves multiple participants and requires preparing meals with food variety and nutritional density, whereas cooking for only one person is prone to dietary monotony (8, 29). Second, social interaction during meals is positively associated with eating motivation and is linked to a reduced prevalence of the “anorexia of aging” that is frequently exacerbated by the psychological burden of loneliness. For community-dwelling older adults, particularly those residing in solitary households, the presence of dining companions is linked to prolonged meal duration and higher total food volume (16, 46). Third, the social environment provides a platform for informal supervision and encouragement; family members or companions are often linked to seniors avoiding nutrient-poor, high-sugar convenience foods, and choosing nutrient-dense options like fruits and vegetables (47–49). As the prevalence of empty-nest and solitary households continues to rise globally, the role of communal dining in relation to lower nutritional risks has transitioned from a social preference to a critical health necessity (7, 19). Our analysis specifically identified a significant deficit in meat and seafood intake among solitary eaters. Group dining settings increase the frequency of animal-source protein provision (28, 34, 50), and the presence of others creates a “social modeling” encouraging the consumption of more diverse and protein-rich dishes (31, 35, 37). The lack of significant differences in fruit and vegetable intake suggests that the consumption of these foods may be more heavily influenced by individual health literacy and geographic food accessibility than by the immediate dining environment (29, 51). The high heterogeneity observed in the intake of fruits, milk, and dairy products likely reflects regional disparities in agricultural supply chains and cultural dietary preferences that override the influence of dining companionship. Thus, targeted intervention strategies need to be developed in accordance with these nuanced dietary characteristics (52, 53).

The positive association of commensality on macronutrient profiles is most pronounced in dietary fat intake. Solitary eaters exhibited significantly lower fat intake, a finding that correlates with the specific composition of solitary meals. In communal settings, the preparation of multiple dishes often incorporates a higher volume of oils and fats, and the inclusion of animal-source proteins, which are naturally fat-rich is more frequent than in simplified solitary meals (34, 50). The presence of companions can create a social settings predisposing older adults to higher-fat dietary choices, consequently correlating with higher total macronutrient density, reflecting the phenomenon of “passive overconsumption” (31, 35, 37). In contrast, the relative stability of carbohydrate intake across dining patterns, particularly in East Asian cohorts (China, Japan, South Korea), points to a cultural dietary inertia where staple food consumption remains a physiological and cultural constant regardless of social context (7, 16, 36). Ultimately, these macronutrient variations underscore how communal dining corresponds to a food composition to favor a more calorically dense and nutrient-rich profile.

The higher total energy intake observed in communal dining may be linked to both psychological and environmental factors. First, the positive dining atmosphere and social engagement inherent in group eating can effectively extend the duration of a meal, decreasing the likelihood of premature termination and thus maximizing caloric consumption (46, 54). Second, the structural variety of communal meals prevents the simplification trap where solitary older adults, deterred by the effort of complex preparation, default to low-energy, single-item snacks (8, 29). Additionally, the presence of peers provides emotional buffer that can alleviate mealtime stress and counter the appetite-suppressing effects of social isolation (16, 46). These findings highlight communal dining as a high-potential, non-pharmacological intervention for addressing geriatric energy deficiency.

The significant association between solitary eating and elevated depression risk may be potentially explained by the erosion of social interaction during mealtimes (9). As a primary medium for emotional reciprocity and social cohesion, commensality correlates with a stronger sense of belonging and psychological security (55). In contrast, chronic solitary eating is concurrently observed with social isolation, which presents a strong association with a neuro-psychological cascade associated with depressive symptoms and subsequently correlates with lower social appetite and a self-reinforcing cycle of decline (42, 43). Our subgroup analysis by gender revealed a marginally significant association, yet this masks profound internal heterogeneity. Some studies, such as Wang et al. (42), report uniform risk across genders, but others like Son et al. (38) find a disproportionately high risk in females. This suggests that the psychological association of dining alone is context-dependent, shaped by cultural norms, traditional family roles, and the availability of localized support networks rather than mere biology (7, 9). Furthermore, dinner appears to be the most critical mealtime for social health; in most cultures, evening meals are the primary site for familial and social bonding, which makes solitary dinner a particularly potent correlate of loneliness and psychological distress (43, 56).

The presence of moderate to high heterogeneity in several of our pooled analyses, particularly regarding dietary intake and depression risk (*I*^2^ = 82.6%), carries substantial methodological and practical implications. Rather than diminishing the validity of our findings, this heterogeneity highlights that the relationship between dining patterns and geriatric health outcomes is highly context-dependent, rather than uniform across global cohorts. Clinically, these variations underscore that a “one-size-fits-all” communal dining strategy may be inadequate. Instead, public health interventions must be tailored to account for regional variations in dietary measurement tools, localized social support architectures, and cultural expectations surrounding mealtime companionship, which our subgroup analyses identified as major drivers of the observed variance. Decoupling these contextual layers will be essential for translating these aggregated associations into effective, individualized geriatric care policies.

Our analysis suggested a heightened sensitivity in Japanese populations, potentially reflecting the unique cultural emphasis on group harmony. In contrast, the results for the Chinese demographic are derived from a single large-scale study and require cautious interpretation and further validation in diverse regional contexts. Given that the vast majority of our sample is concentrated in East Asia (16 of 21 studies), these findings may heavily reflect Confucian traditions and dietary collectivism, where communal meals carry high emotional weight. Consequently, the generalizability of our findings to Western societies warrants considerable caution. In collectivistic East Asian cultures, the transition to solitary dining often represents a profound disruption of social networks deeply tied to familial filial piety and emotional reciprocity, thereby potentially exacerbating the risk of psychological distress. Furthermore, East Asian dietary structures, characterized by a high adherence to shared staple foods and multiple communal side dishes, inherently facilitate dietary diversification during social dining. In contrast, in Western populations where individualized plating and solitary convenience meals are more culturally institutionalized, the nutritional deficits associated with eating alone might operate through distinct socioeconomic or functional pathways rather than purely social facilitation. Therefore, our pooled estimates should be interpreted primarily as an observational baseline for collectivistic aging societies. Future multicenter studies across different global regions countries are needed to validate the observed cultural variances and assess their generalizability in Western societies.

The contemporary socio-demographic shift underscores the critical public health imperative of these findings. As the traditional multi-generational family structures continue to fragment along with population aging, solitary living has reached unprecedented prevalence (57, 58). This transition is often observed following bereavement or the geographic dispersal of adult children. As a result, for millions of seniors, solitary eating is not a matter of personal preference but a structural reality (59, 60). In the post-pandemic landscape, the digital divide and habitual social withdrawal have further eroded the traditional communal dining experience (61, 62). These trends indicate that the adverse associations between solitary eating and health, including malnutrition and social isolation, are becoming systemic challenges (30, 63). Addressing the nutritional and psychological risks of solitary dining thus represents an urgent frontier for geriatric health policy.

Several limitations of this review warrant consideration. First, the predominantly cross-sectional nature of the included studies precludes definitive causal inference, primarily due to the lack of temporal sequence. This makes it impossible to rule out reverse causality, where pre-existing depressive symptoms or nutritional inadequacy drive older adults to social withdrawal and solitary dining. Additionally, to capture the natural and long-term eating behaviors of community-dwelling older adults and minimize recall bias, RCTs and case-control studies were excluded. Second, the substantial heterogeneity in dietary assessment methods and depression scales may limit the direct comparability of effect sizes across different populations. Third, residual confounding may persist stemming from inconsistent adjustment for socio-cultural factors such as socioeconomic status or pre-existing comorbidities. Crucially, the potential conflation of living alone with eating alone remains a measurement risk, although we prioritized studies focusing on the behavioral act of eating. Future research must utilize more granular metrics, such as a cross-analysis of meal frequencies and household size, to decouple the specific association of solitary dining from broader social isolation.

In conclusion, our analysis revealed that the eating environment is closely tied to geriatric health. Commensality is significantly associated with increased intake of high-quality protein and fats, and chronic solitary eating is accompanied by elevated depression. The psychological association of solitary eating is highly sensitive to mealtime and social context, with solitary dinner posing the highest risk. Commensality appears to safeguard against nutritional inadequacy and psychological distress through the integrated mechanisms of social facilitation and dietary diversification. To promote geriatric resilience and integrate communal dining into holistic geriatric health policies, future research should prioritize longitudinal cohort designs and interventional trials to establish causality, with a specific focus on the role of companion characteristics and meal frequency.

## Data Availability

The original contributions presented in the study are included in the article/Supplementary material, further inquiries can be directed to the corresponding authors.
