# Supplementary material for: The association of communal vs. solitary dining on nutritional intake and depression risk in older adults: a systematic review and meta-analysis
Source: Front Nutr. 2026 Jul 13;13:1878848. doi: 10.3389/fnut.2026.1878848 (PMC13402156; doi:10.3389/fnut.2026.1878848)
Supplement: Supplementary file 1 [file Table_1.docx]

Supplementary Material

**Table S1** Search strategy on PubMed

| **Item** | **Search strategy** |
| --- | --- |
| #1 | ((((elderly) OR ("older adult")) OR (senior)) OR (aged)) OR (geriatric) |
| #2 | (((((((((((("eating alone") OR ("meal alone")) OR ("dining alone")) OR ("solo dining")) OR ("individual dining")) OR ("Solo eating")) OR ("eating together")) OR ("meal together")) OR ("dine together")) OR ("solitary eating")) OR (commensality)) OR ("shared meal")) OR ("eating with others") |
| #3 | (((((((((((("Food Intake*") OR ("nutrient intake*")) OR ("energy intake*")) OR ("feed intake*")) OR ("Nutritional Intake*")) OR (depress*)) OR ("mental health")) OR ("psychological status") OR ("Depressive Symptom*")) OR ("Emotional Depression")) OR ("depressive state")) OR ("depressive syndrome")) |
| #4 | #1 AND #2 AND #3 |

**Table S2** Search strategy on Embase

| **Item** | **Search strategy** |
| --- | --- |
| #1 | 'elderly':ab,ti OR 'older adult':ab,ti OR 'senior':ab,ti OR 'aged':ab,ti OR 'geriatric':ab,ti |
| #2 | 'eating alone':ab,ti OR 'meal alone':ab,ti OR 'dining alone':ab,ti OR 'solo dining':ab,ti OR 'individual dining':ab,ti OR 'Solo eating':ab,ti OR 'eating together':ab,ti OR 'meal together':ab,ti OR 'dine together':ab,ti OR 'solitary eating':ab,ti OR 'commensality':ab,ti OR 'shared meal':ab,ti OR 'eating with others':ab,ti |
| #3 | 'Food Intake*':ab,ti OR 'nutrient intake*':ab,ti OR 'energy intake*':ab,ti OR 'feed intake*':ab,ti OR 'Nutritional Intake*':ab,ti OR 'depress*':ab,ti OR 'mental health':ab,ti OR 'psychological status':ab,ti OR 'Depressive Symptom':ab,ti OR 'Emotional Depression':ab,ti OR 'depressive stat':ab,ti OR ' depressive syndrome':ab,ti |
| #4 | #1 AND #2 AND #3 |

**Table S3** Search strategy on Cochrane Library

| **Item** | **Search strategy** |
| --- | --- |
| #1 | (elderly OR older adult OR senior OR aged OR geriatric) |
| #2 | (eating alone OR meal alone OR dining alone OR individual dining OR solo dining OR Solo eating OR eating together OR meal together OR dine together OR solitary eating OR commensality OR shared meal OR eating with others) |
| #3 | (Food Intake* OR nutrient intake* OR energy intake* OR feed intake* OR Nutritional Intake* OR depress* OR mental health OR psychological status OR Depressive Symptom* OR Emotional Depression OR depressive state OR depressive syndrome) |
| #4 | #1 AND #2AND #3 |

**Table S4** Search strategy on Web of Science

| **Item** | **Search strategy** |
| --- | --- |
| #1 | elderly (All Fields) or "older adult" (All Fields) or senior (All Fields) or geriatric (All Fields) |
| #2 | "eating alone" (All Fields) or "meal alone" (All Fields) or "dining alone" (All Fields) or "individual dining" (All Fields) or "solo dining" (All Fields) or "eating together" (All Fields) or "meal together" (All Fields) or "dine together" (All Fields) or " solitary eating " (All Fields) or commensality (All Fields) or "shared meal" (All Fields) or "eating with others" (All Fields) |
| #3 | "Food Intake*" (All Fields) or "nutrient intake*" (All Fields) or "energy intake*" (All Fields) or "feed intake*" (All Fields) or "Nutritional Intake*" (All Fields) or depress* (All Fields) or "mental health" (All Fields) or "psychological status" (All Fields) or "Depressive Symptom*" (All Fields) or "Emotional Depression" (All Fields) or "depressive state" (All Fields) or "depressive syndrome" (All Fields) |
| #4 | #1 AND #2AND #3 |

**Subgroup Analysis**


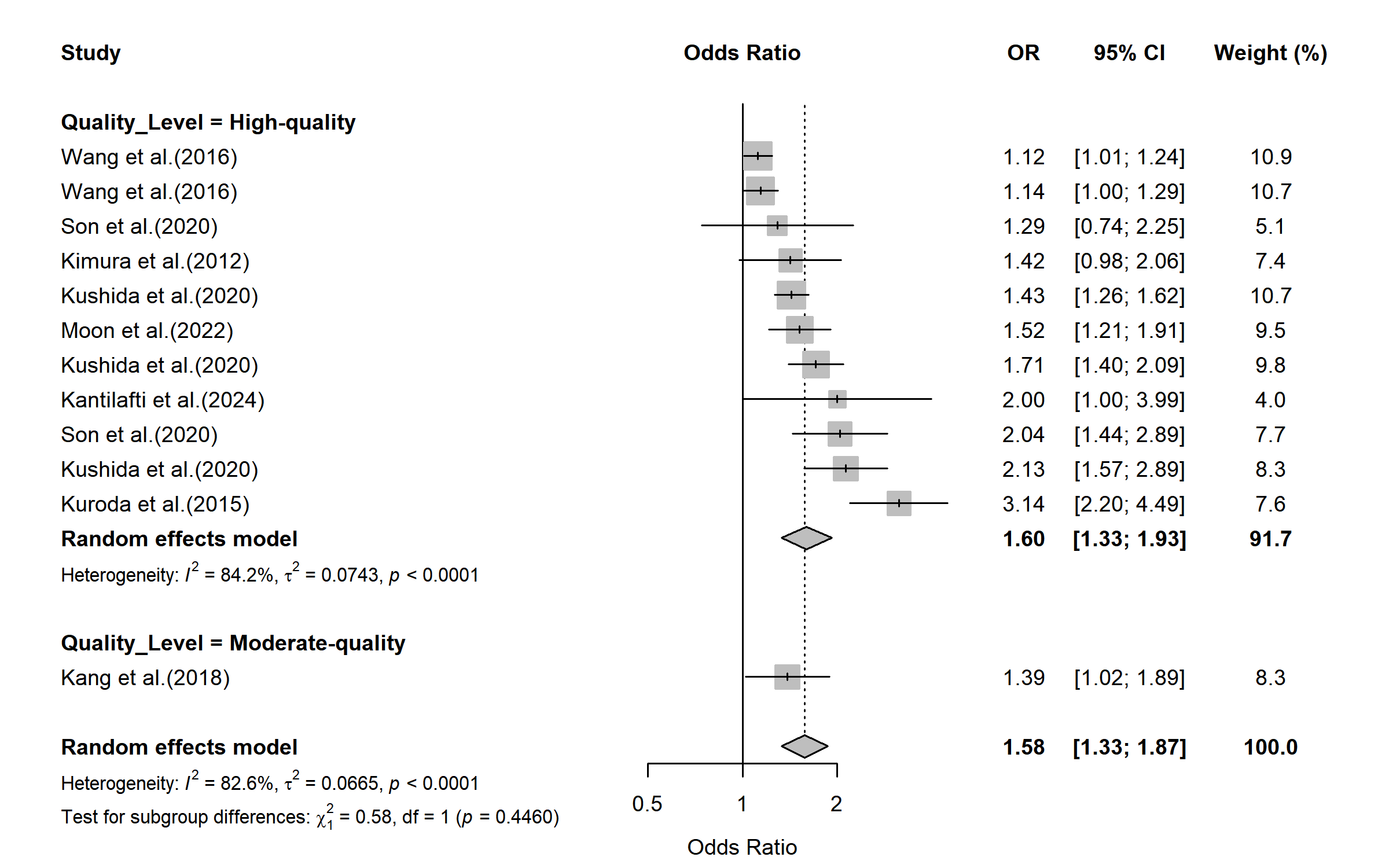


**Figure S1** Subgroup analysis of psychological status by study quality


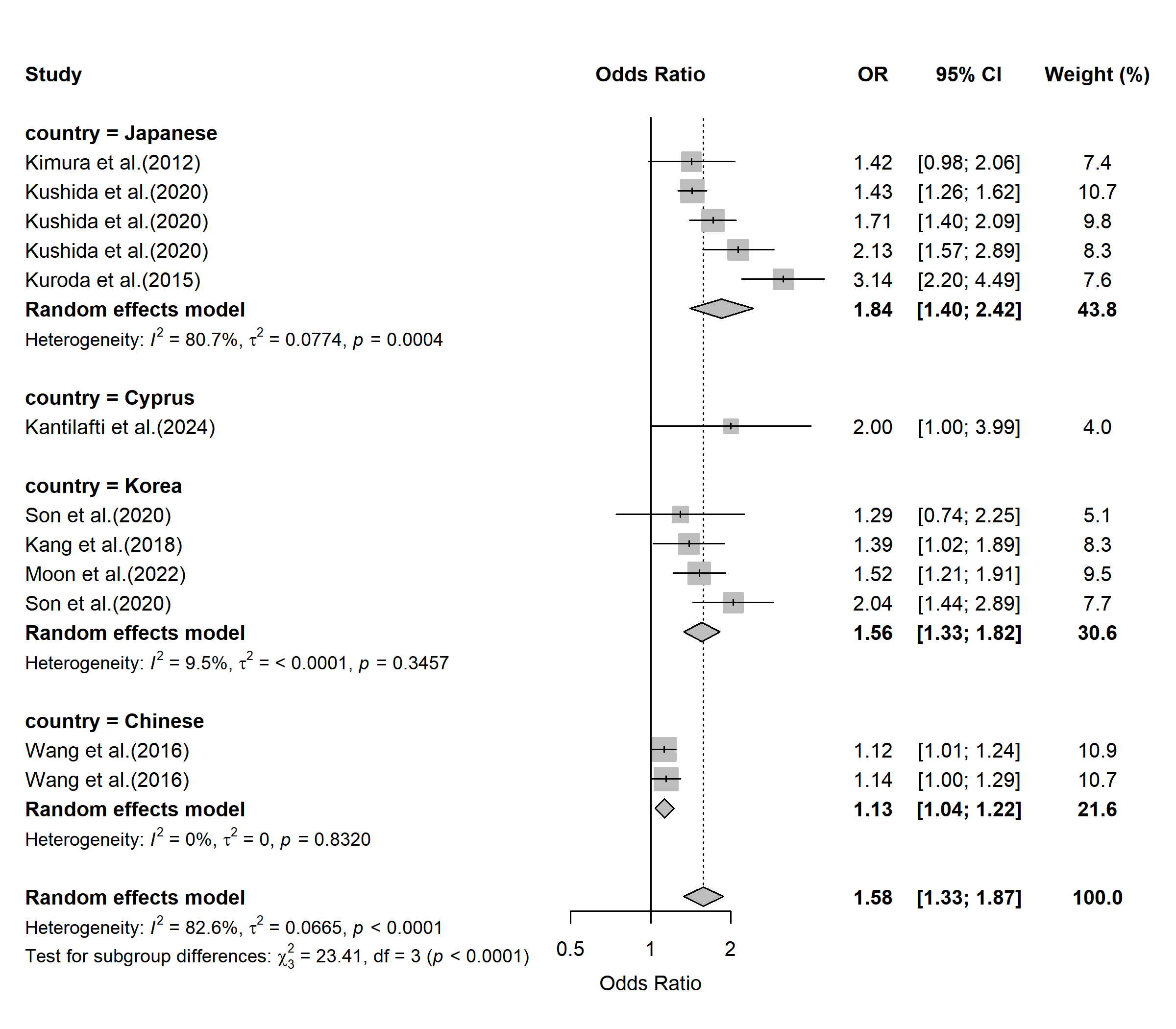


**Figure S2** Subgroup analysis of psychological status by country

**
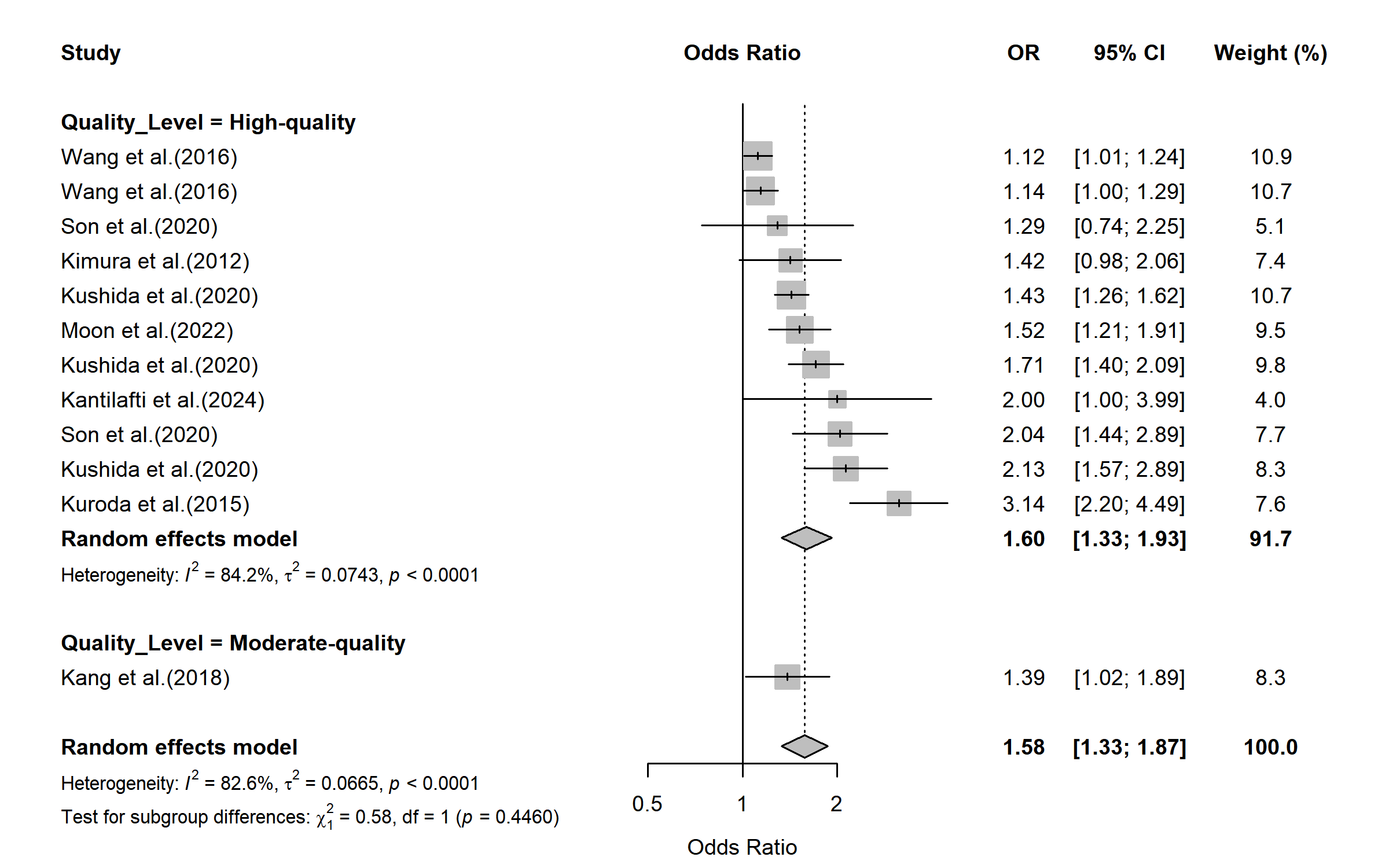
**

**Figure S3** Subgroup analysis of psychological status by assessment scale

**Sensitivity Analysis**

**
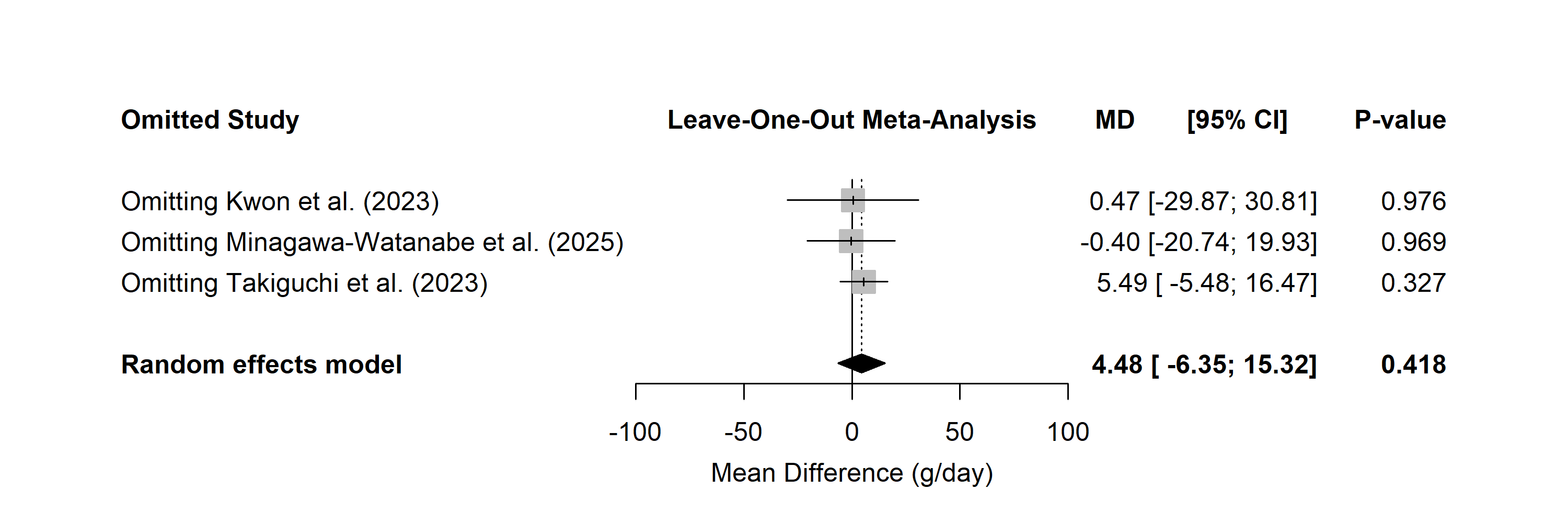
**

**Figure S4** Sensitivity analysis: cereals and tubers

**
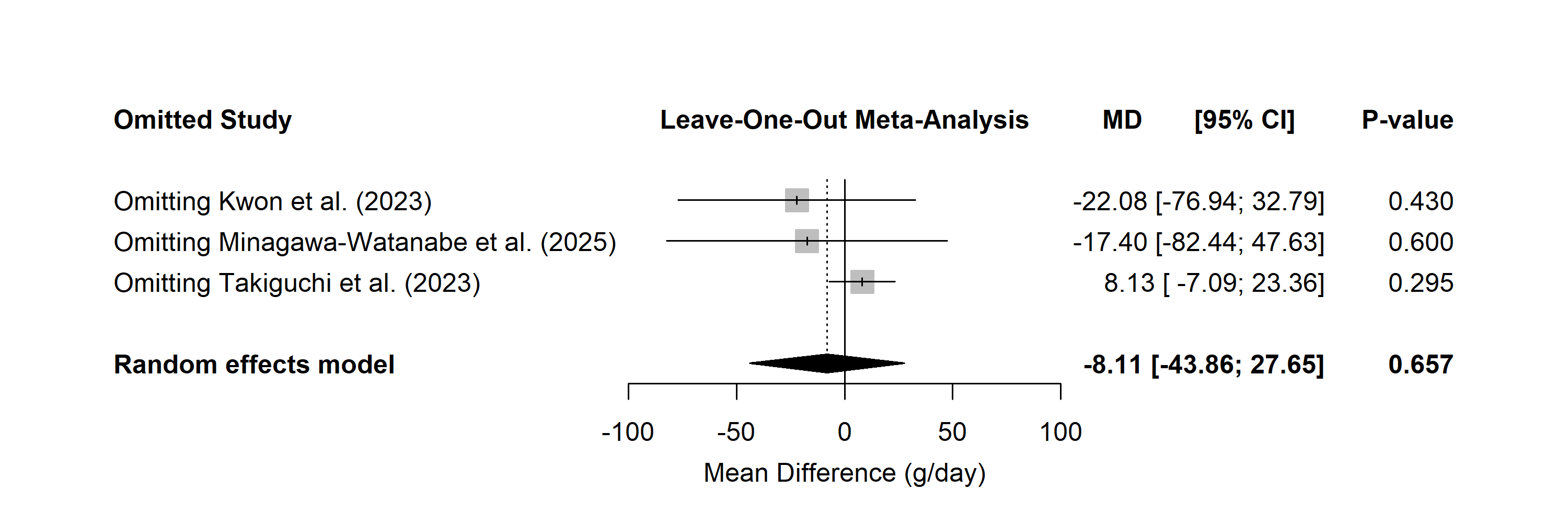
**

**Figure S5** Sensitivity analysis: fruit

**
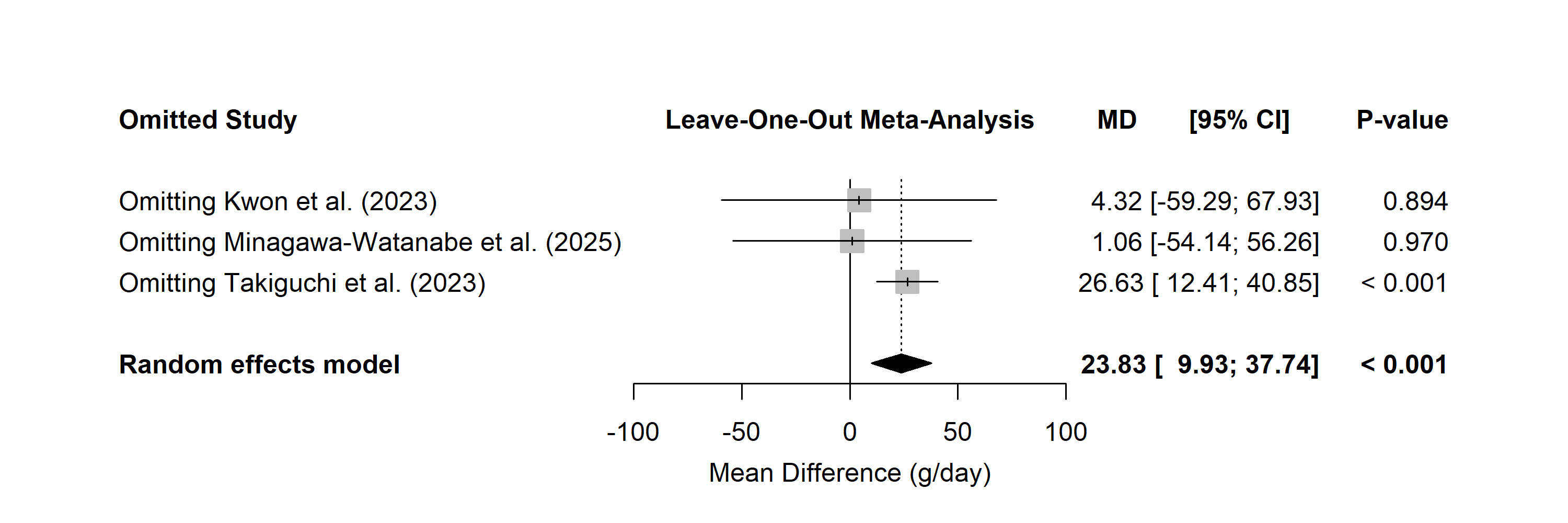
**

**Figure S6** Sensitivity Analysis: Vegetables


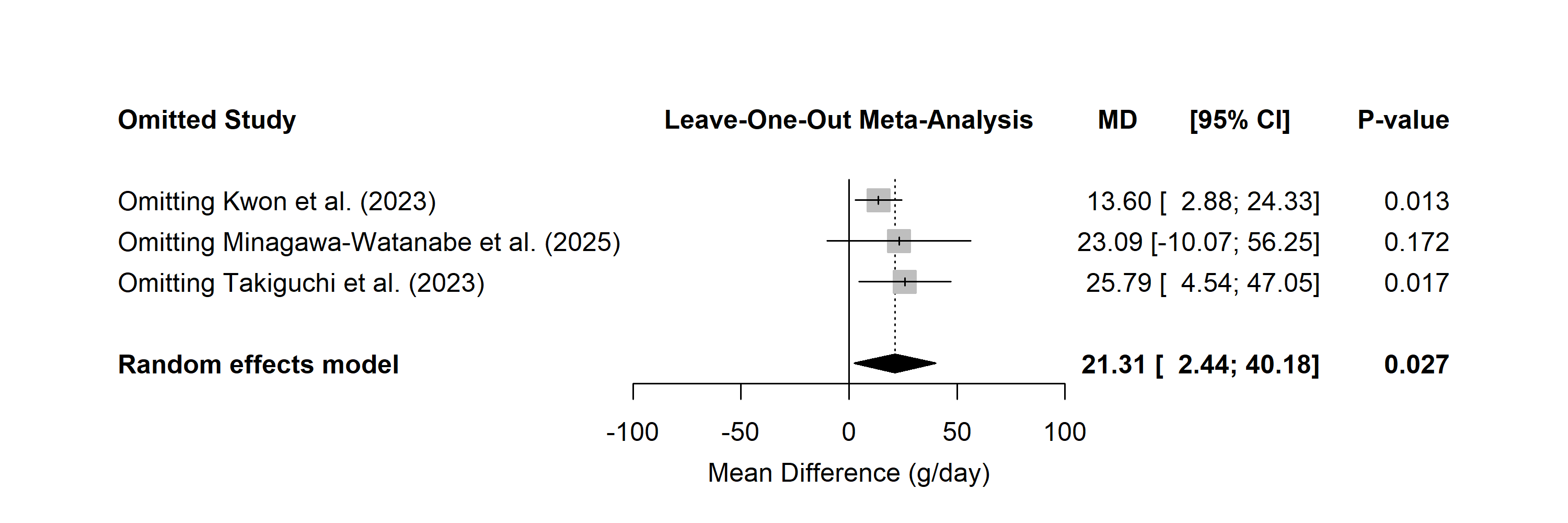


**Figure S7** Sensitivity analysis: meat and seafood


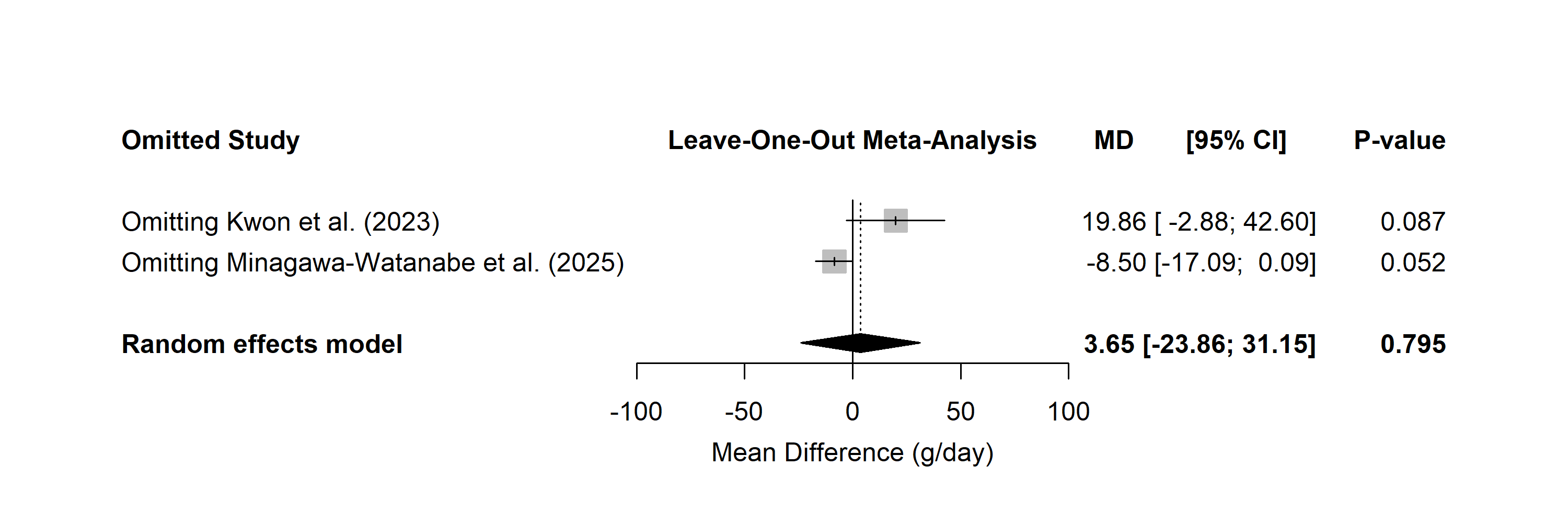


**Figure S8** Sensitivity analysis: milk and dairy products


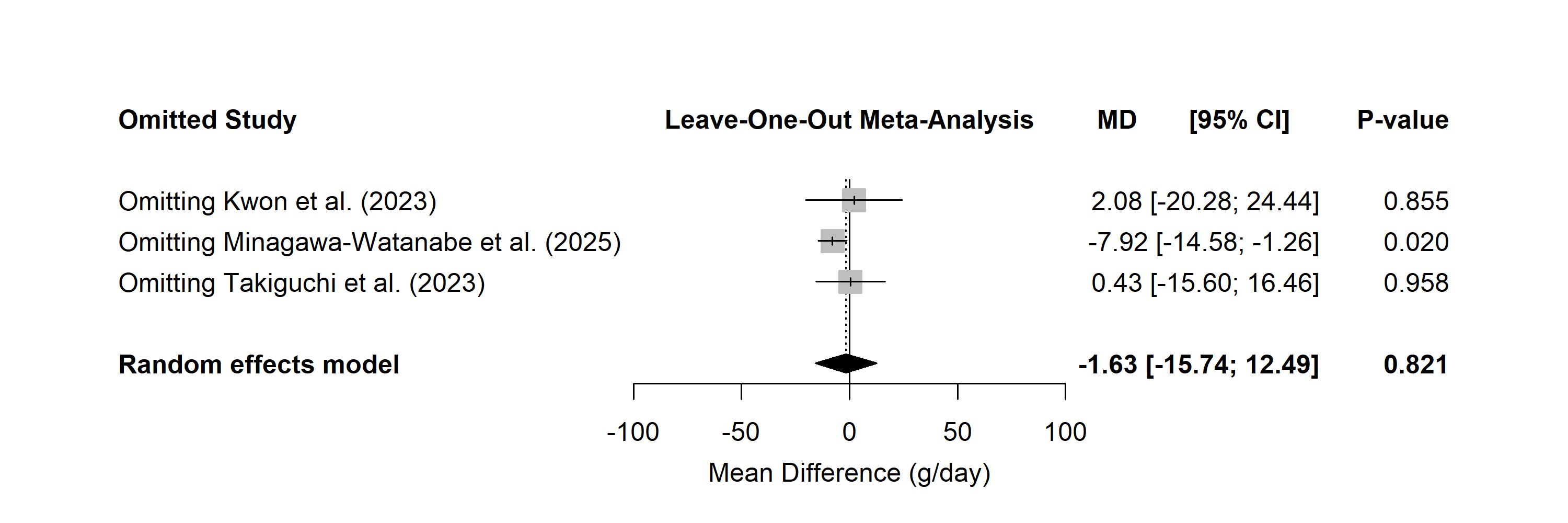


**Figure S9** Sensitivity analysis: soybeans


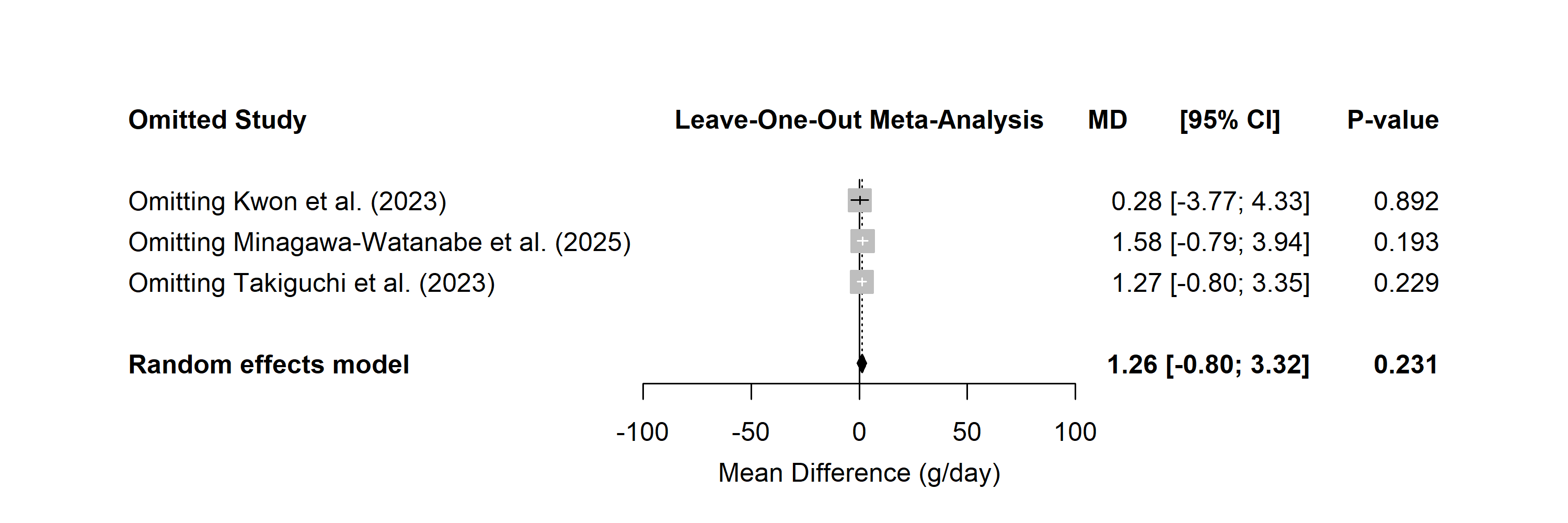


**Figure S10** Sensitivity analysis: eggs


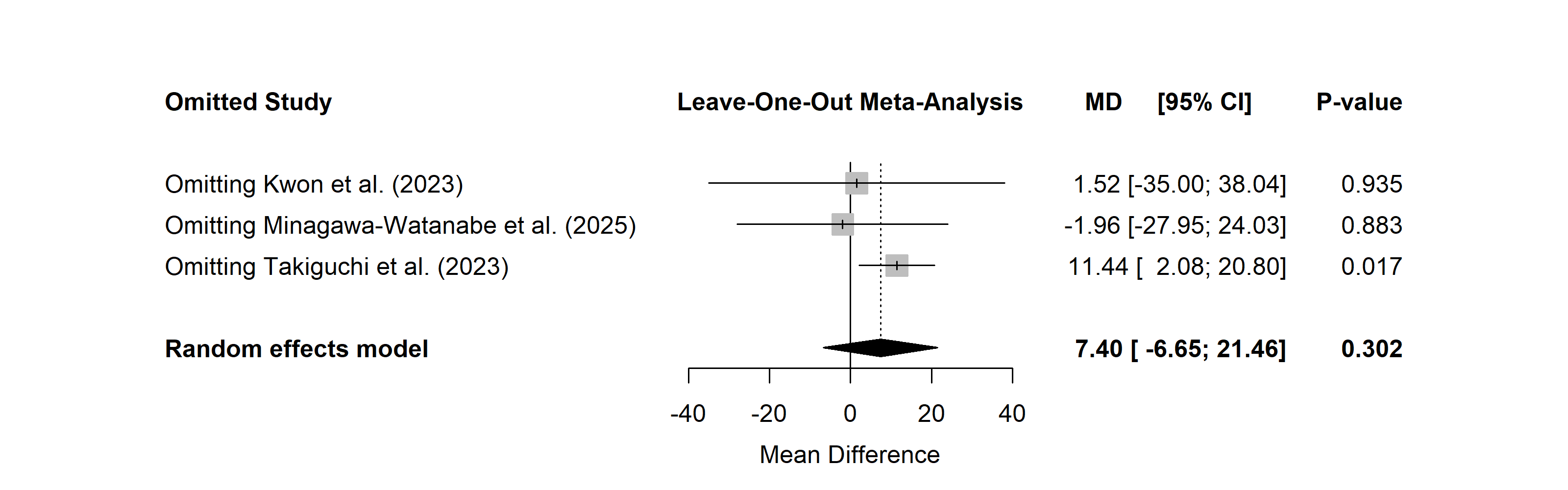


**Figure S11** Sensitivity analysis: carbohydrate


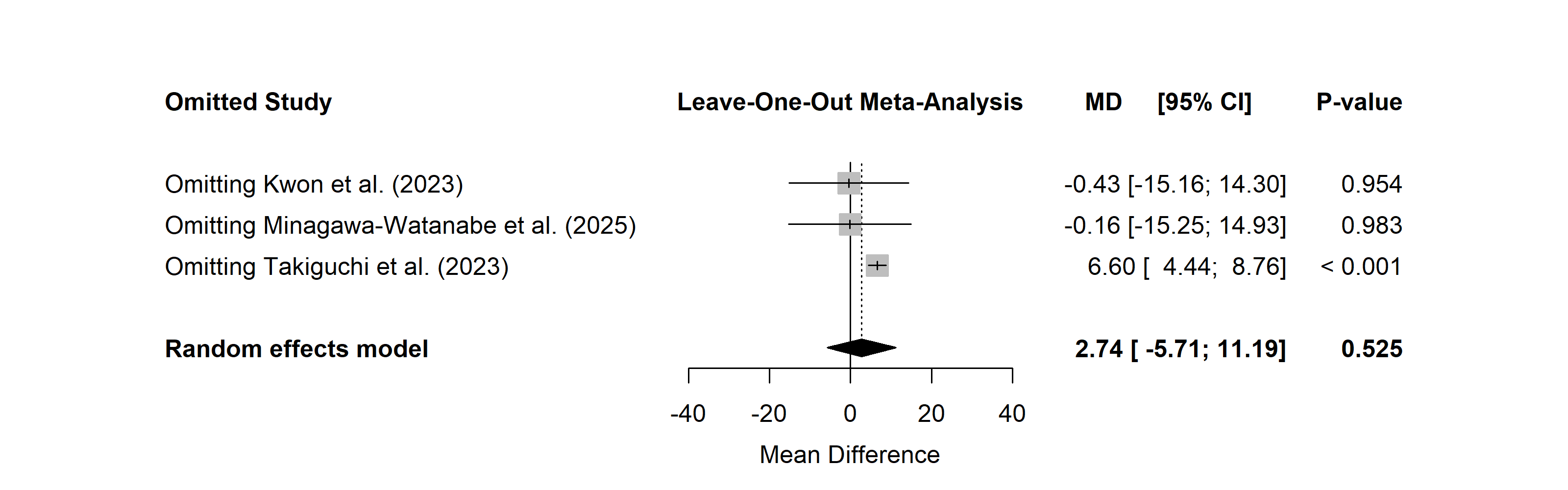


**Figure S12** Sensitivity analysis: protein


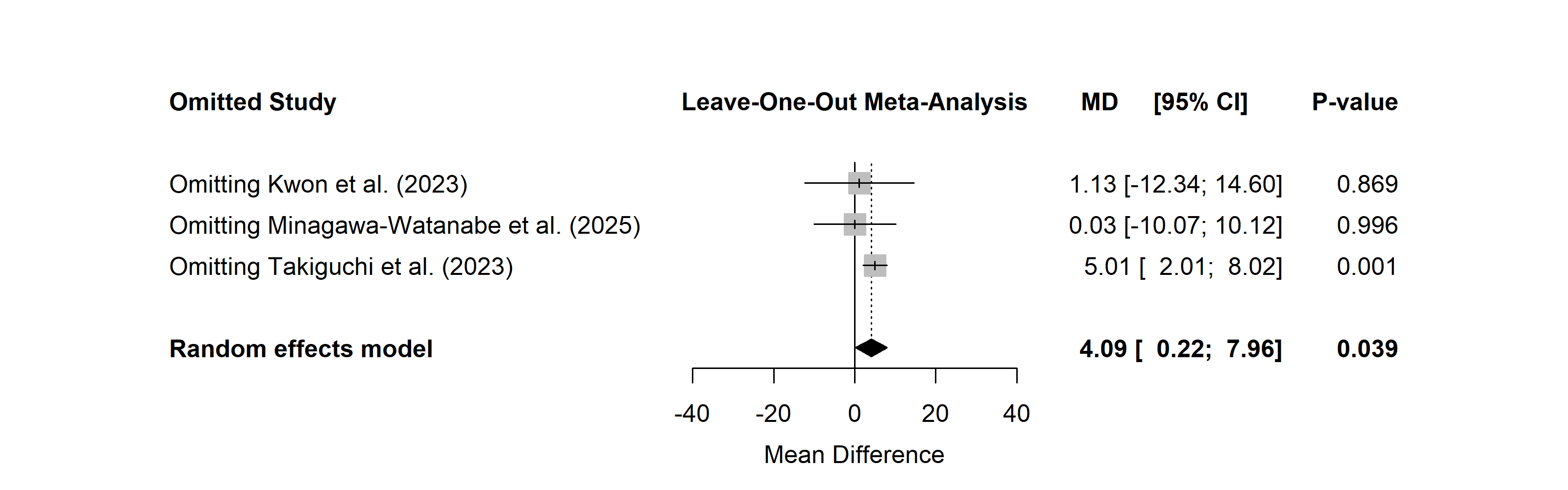


**Figure S13** Sensitivity analysis: fat


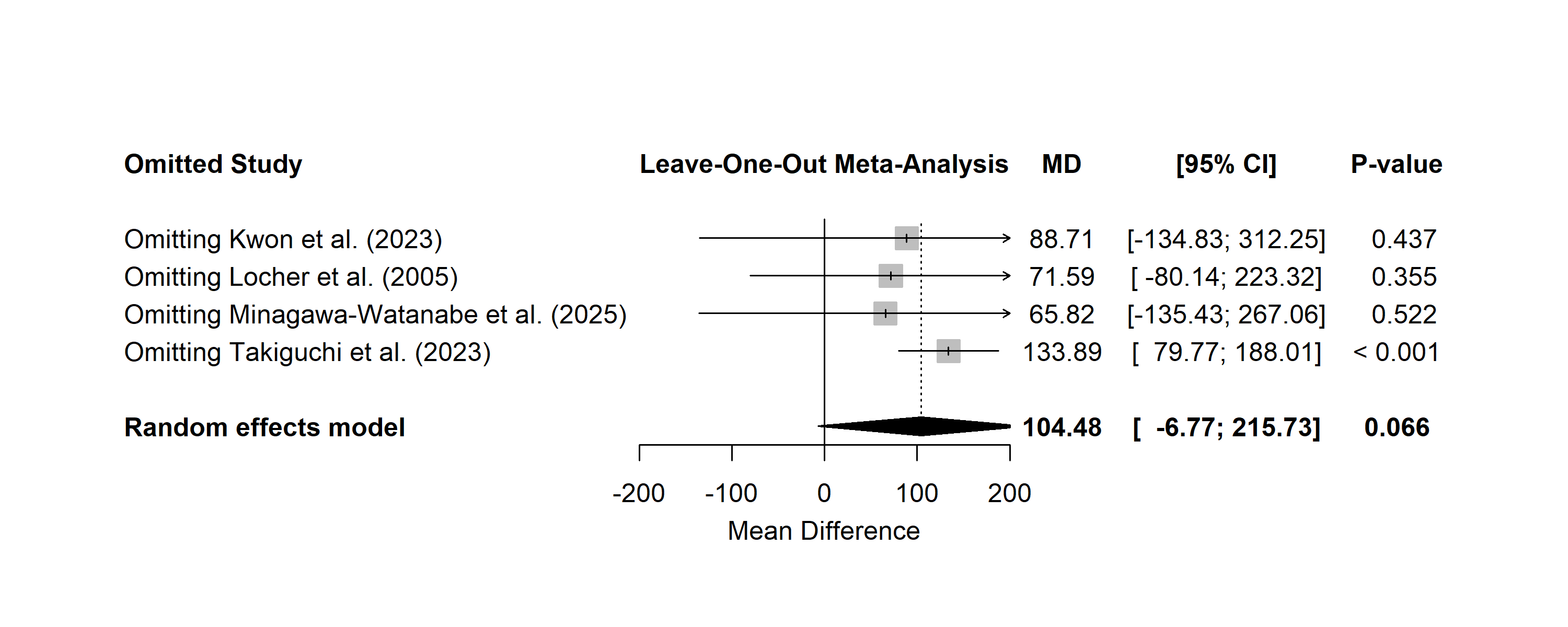


**Figure S14** Sensitivity analysis: total energy


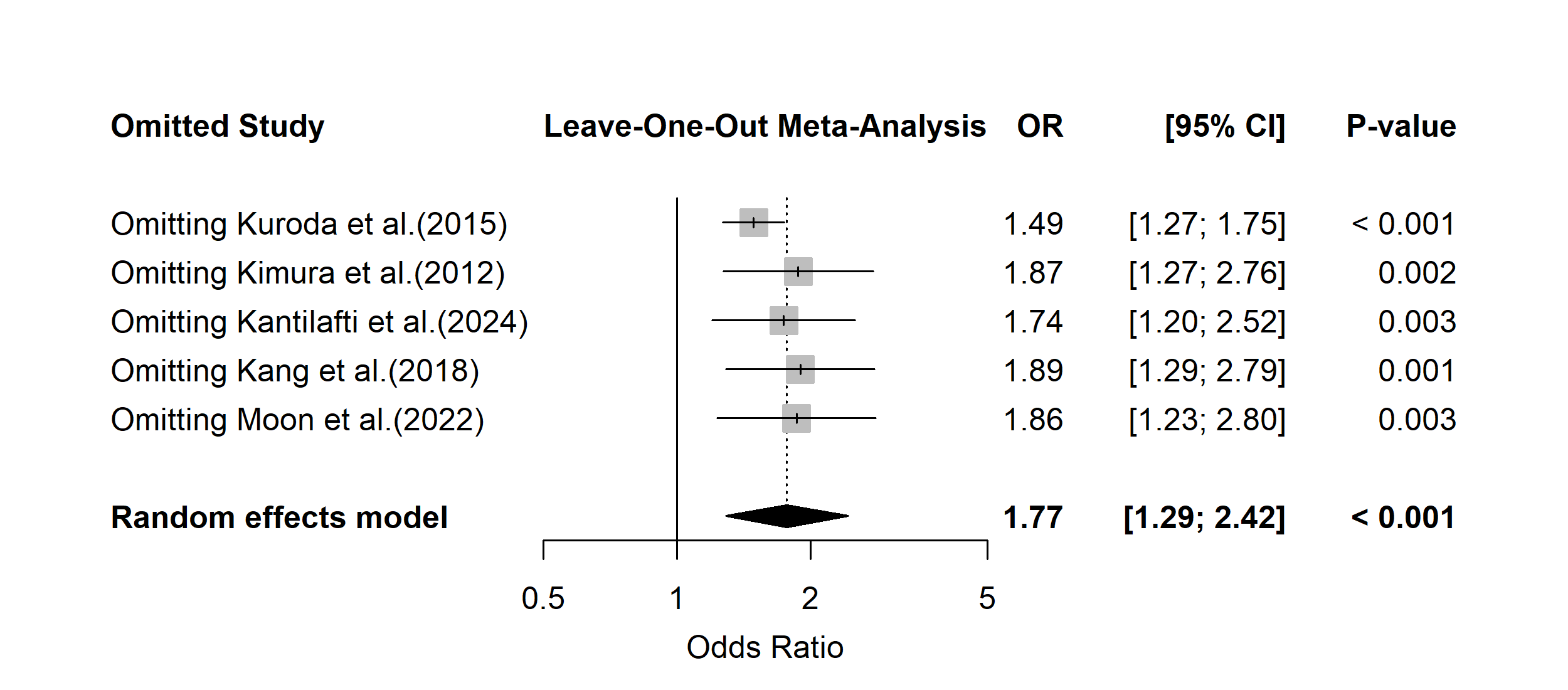


**Figure S15** Sensitivity analysis: psychology status
